# Perspectives of health care professionals’ on delivering mHealth sexual and reproductive health services in rural settings in low-and-middle-income countries: a qualitative systematic review

**DOI:** 10.1186/s12913-022-08512-2

**Published:** 2022-09-09

**Authors:** Alexander Suuk Laar, Melissa L. Harris, Desalegn Markos Shifti, Deborah Loxton

**Affiliations:** 1 The University of Newcastle, Australia, School of Public Health and Medicine, Centre for Women’s Health Research, Faculty of Health and Medicine, Hunter Medical Research Institute, Callaghan, New South Wales 2308 Australia; 2 REJ Institute, Research and ICT Consultancy Services, Tamale, Ghana

**Keywords:** Health care professionals, Mobile phones, mHealth, Sexual and reproductive health, Information and services, Low-and middle-income countries

## Abstract

**Background:**

In low to middle income countries (LMICs) with limited health care providers (HCPs) and health infrastructure, digital technologies are rapidly being adopted to help augment service delivery. In this sphere, sexual and reproductive health (SRH) services are increasingly leveraging mobile health (mHealth) technologies to improve service and information provision in rural areas. This systematic review aimed to identify HCPs perspectives on barriers to, and facilitators of, mobile phone based SRH services and information in rural areas of LMICs from current literature.

**Methods:**

Searches were conducted using the following databases: Medline, Scopus, PsychINFO, CINAHL and Cochrane Library. Based on the inclusion and exclusion criteria, twelve full text qualitative studies published in English between January 2000 and December 2020 were included. The methodological quality of papers was assessed by two authors using the critical skills appraisal programme and synthesized using the narrative thematic analysis approach.

**Results:**

Positive HCPs experiences surrounding the provision of mHealth based SRH services in LMICs included saving consultation time, ability to shift tasks, reduction in travel costs, easy referrals and follow up on clients, convenience in communicating health information confidentially, and the ability to consult groups of clients remotely rather than face-to-face. Barriers to the provision of mHealth reported by HCPs included lack of technological infrastructure, unreliable networks, limited power, the cost of mobile airtime/data and mobile phones and limited technological literacy or skills.

**Conclusions:**

Implementing innovative mHealth based SRH services could bridge a service provision and access gap of SRH information and services in rural areas of LMICs. Despite the advantages of this technology, several challenges associated with delivering mHealth SRH services need to be urgently addressed to enable scale-up and integration of sexual and reproductive mHealth into rural health systems.

**Supplementary Information:**

The online version contains supplementary material available at 10.1186/s12913-022-08512-2.

## Background

Low-and-middle-income countries (LMICs), where half of the world’s population currently live [1], generally lack access to quality health including reproductive health services [1, 2]. Reproductive health is a state of complete physical, mental and social well-being and not merely the absence of disease or infirmity, in all matters relating to the reproductive system and its processes [3]. Reproductive health hence implies that both men and women are able to have a satisfying and safe sex life and that they have the capability to reproduce and the freedom to decide if, when, and how often to do so [3]. In LMICs, despite the importance of sexual and reproductive health (SRH) information, SRH education programs do not currently reach most rural people. Further, services for contraception, family planning and sexually transmitted infections (STIs) are frequently lacking, especially in rural settings [4]. Evidence suggests that in rural areas in LMICs, increasing access to and use of SRH information and services can reduce unsafe sexual behaviour [3, 4].

Lack of access to and use of essential SRH information and services by rural populations in LMICs is largely related to cultural, social and psychosocial factors [5–7], lack of health care providers (HCPs) and health infrastructure resulting in long distance and cost of transportation and healthcare services [1, 8, 9] compared to urban areas [9, 10]. All these factors together contribute to a high unmet need for SRH information and services leading to poor health outcomes [11] such as unintended pregnancy, STIs including HIV, and increased maternal morbidity and mortality [12–14]. Thus evidence based innovative interventions that might meet rural populations’ SRH needs is vital in the context of LMICs [15].

In LMICs, digital health technologies have been introduced into rural health services [16–18]. Reproductive health programs are also leveraging innovative mobile health mHealth technologies for improving quality and access to SRH information and services for populations residing in rural areas [19–21] (regions with population densities of fewer than 150 people per square kilometre and more than 50% of the population living in areas classified as rural communities with poor access to medical care and health professionals [22]. Mobile health technologies interventions are cost-effective in engaging poor rural people with a range of SRH information and services in LMICs [17–19, 23, 24]. The World Health Organization has underscored the importance of improving SRH of rural populations by providing accessible, acceptable and affordable SRH information and services via mHealth technologies [25].

There is growing evidence for providing mHealth based SRH information and services to people in the rural context in LMICs [18, 26–29]. However, evidence on factors that influence access to mobile phone based SRH information and services to rural population and youth in LMICs is limited. Identifying barriers and facilitators for providing access to mobile phone based SRH information is vital for improving services that meet rural population needs [30, 31]. The current study therefore reviewed existing literature where the perspectives of HCPs in implementing mHealth SRH services for populations in rural areas of LMICs had been explored. Specifically, we explored HCPs experiences on barriers and facilitators in the delivery of mobile phone based SRH information and services to rural populations including young people in rural settings of LMICs.

## Methods

### Protocol and registration

This systematic review followed the preferred Reporting Items for Systematic Reviews and Meta-Analyses (PRISMA) guidelines [32]. It was registered with PROSPERO on October 23, 2020 (Prospero Number: CRD42020210777).

### Database search

We developed a search strategy for each database using the guidelines developed by the Cochrane Qualitative Research Methods Group for searching qualitative evidence [33]. A systematic search of six online journal databases was carried out to find relevant mHealth studies in the context of LMICs. Searches were limited to studies published in English from January 2000 to December 2020 as the field of mHealth has emerged over the last two decades [34].

Five domains were searched: “mHealth intervention provider”, “mHealth platforms”, “mHealth intervention recipient,” “mHealth intervention services” and “geographical setting (LMICs)” (see Table 1).

**Table 1:** Search terms

Search domains	Search Terms
**mHealth intervention provider**	healthcare providers, lay health workers, health counsellors, healthcare workers, health educators
**mHealth platforms**	mobile health, mHealth, mobile phone health technology, mobile phone health, digital mobile health, digital mobile phone health
**mHealth intervention recipient**	Women, men, adult men adult women, young, adolescent, young people, youth population, young women, young girls, young boys, young men, young women and men, young girls and boys, adolescent girls, adolescent boys, adolescent girls and boys
**mHealth intervention services**	reproductive health, sexual health, sexually transmitted infections such as HIV, contraception and family planning, family planning information and services
**Geographic setting**	low-income countries, low-and-middle-income countries

### Search terms

The first author (ASL) developed the search terms which were reviewed by MLH and DL. The search terms were then refined in consultation with the College of Health and Wellbeing’s librarian. Search terms were combined with an “OR” Boolean operator, and terms between each domain were linked with “AND” operators.

### Eligibility criteria

#### Inclusion and exclusion criteria

We included studies that reported on mHealth interventions which included SRH information on contraception, family planning, HIV and STIs prevention for people in rural settings in LMICs (classified using World Bank classifications) [35]. Studies that were not peer-reviewed, for example, conference presentations, student theses, editorials, review articles, letters to the editor, commentaries, and symposium proceedings, were excluded.

#### Data sources and search strategy

We searched six databases (Medline, Scopus, PsychINFO, CINAHL and Cochrane Library) for published literature in English that reported on mHealth SRH intervention delivery barriers and facilitators for people in rural settings in LMICs. In addition to these sources, reference lists of all included studies and key references of relevant systematic reviews on mHealth studies available as well as Google were searched to identify any further relevant articles. The search terms which were used to perform Medline search strategy are shown in Table 2. Search strategies for the remaining databases are included in an online supplement.

**Table 2:** Medline Search Strategy

healthcare providers* OR Healthcare professionals* OR health provider* OR health counsellor* OR health educator* AND mobile health* OR mHealth* OR mobile phone health technology* OR mobile phone health* OR digital mobile health* OR digital mobile phone health* OR voice messaging*OR phone calls* OR voice calls* OR SMS text-messaging* OR short message service* OR IVR calls* OR interactive voice respose calls AND young adult* OR youth* OR adolescent* OR young people* OR youth population* OR young wom?n* OR young girl* OR young boy* OR young m?n* OR young women* OR emerging adult* OR men* OR women* OR adult men* OR adult women* OR adolescent girl* OR adolescent boy* OR adolescen* AND reproductive health* or sexual health* or HIV* or contraception* or contraceptives* or modern contraception* or contracept service* or contracept educat* or contracept counsel*OR family planning AND low-income countries* OR low-and-middle-income nation* OR low to middle income countries* O middle-income countr* OR low resource countries All limit to (english language and full text and humans and yr = “2000 - 2020”)	

#### Data extraction

The search results from the databases were first downloaded into the citation management system (Endnote X9 software )[36] and later imported into the Covidence online platform by the first author (ASL). Duplicates were automatically removed by the Covidence system.

#### Study selection

**Fig. 1:**
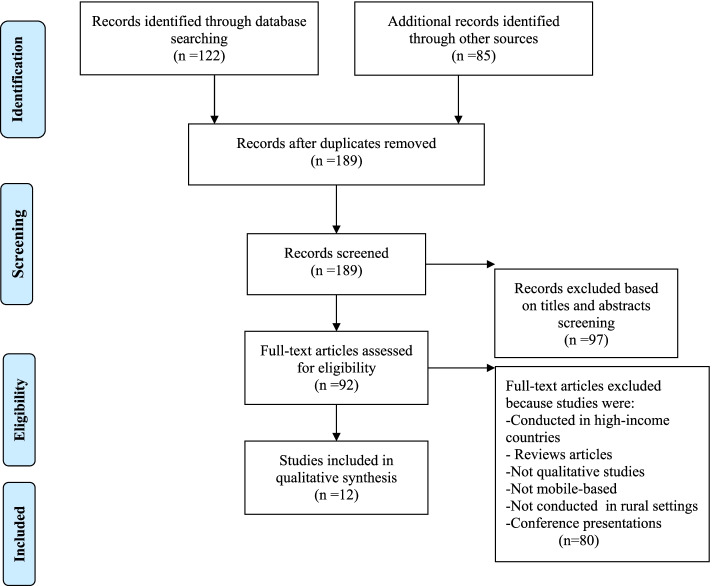
PRISMA Flow diagram

Data extraction to determine the relevancy of the papers was carried out by two authors (ASL and DMS). Following a data extraction form, the two authors independently read all included articles based on study author, year of publication, description of the study context, study methods, study population, mHealth intervention services, mHealth platforms and study findings. The two authors independently reviewed the full text articles for suitability for the review. At all stages, any discrepancies were discussed until a consensus was reached. A total of 92 full text articles were assessed according to the selection criteria and 12 studies were retained for this qualitative synthesis [29, 37–47]. The authors followed the 2009 Preferred Reporting Items for Systematic Review and Meta Analyses (PRISMA) flow chart [48] to report the study selection process (see Fig. 1).

### Sythensis methods

#### Quality assessment

##### Critical appraisal of included studies

Two authors (ASL and DMS) independently and critically appraised 12 eligible papers for methodological quality using critical appraisal tool for mixed studies review (MSR) [49]. We appraised the studies in line with the presence or absence of a primary qualitative study questions, study design, sampling method, study context, data collection, data analysis, ethical considerations, researchers’ reflexivity, conclusions drawn justified by study findings, transferability of study findings to similar settings (Table 3). The methodological quality of all included studies were assessed based on a ten point question criteria. For each criterion, the presence denoted yes scored as 1 and absence no scored as 0 respectively. The studies were scored using percentages (0-100% with one point representing 10%). The scores ranged from 50 to 100%. They were interpreted as follows: below 50% low quality, 50-75% average quality, and 76-100% high quality (Table 3). The quality score was calculated as [(number of yes responses divided by the number of the relevant criteria (10) × 100]. Based on the scoring system, we retained all 12 primary studies for the review.

**Table 3:** MSR quality appraisal procedures

Study Authors	Quality assessment questions										
	1	2	3	4	5	6	7	8	9	10	Total (n%)
Jahangir et al. [42]	Yes	Yes	Yes	Yes	Yes	Yes	Yes	No	Yes	Yes	9 (90%)
Peprah et al. [46]	Yes	Yes	Yes	Yes	Yes	Yes	Yes	Yes	Yes	Yes	10 (100%)
Braun et al. [37]	Yes	Yes	Yes	Yes	Yes	No	Yes	No	Yes	Yes	8 (80%
Dev et al. [38]	Yes	Yes	Yes	Yes	Yes	Yes	Yes	Yes	Yes	Yes	10 (100%)
LogIe et al. [44]	Yes	Yes	No	yes	No	No	No	No	Yes	Yes	50 (50%)
Ibembe, [41]	Yes	Yes	Yes	Yes	Yes	Yes	Yes	No	Yes	Yes	9 (90%)
Ong et al. [45]	Yes	Yes	Yes	Yes	Yes	Yes	Yes	No	Yes	Yes	9 (90%)
Khatun et al. [43]	Yes	Yes	Yes	Yes	Yes	Yes	No	No	Yes	Yes	8 (80%)
Hirsch-Moverman et al. [40]	Yes	Yes	Yes	Yes	Yes	Yes	Yes	Yes	Yes	Yes	10 (100%)
Jennings et al. [29]	Yes	Yes	Yes	Yes	Yes	Yes	Yes	No	Yes	Yes	9 (90%)
Hampshere et al. [39]	Yes	Yes	Yes	Yes	Yes	Yes	Yes	Yes	Yes	Yes	10 (100)
Chang et al. [47]	Yes	Yes	Yes	Yes	Yes	Yes	Yes	No	Yes	Yes	9 (90%)
Total = 12											

Key

1. Were the objective(s) or question(s) of the research clearly stated?

2. Was a qualitative approach appropriate for the research question?

3. Was the sampling strategy used appropriate and described?

4. Was the study context clearly described?

5. Was the data collection method appropriate and described?

6. Was the data analysis appropriately described?

7. Does the study adequately address potential ethical issues?

8. Does the study adequately address reflexivity issues?

9. Were the conclusions drawn justified by the findings?

10. Are the findings of the study transferable to my own and other settings?

## Results

### Characteristics of included studies

Of the 12 studies meeting the inclusion criteria, ten were conducted in rural areas [29, 37, 38, 40–44, 46, 47], one in rural/urban areas [45] and one rural and peri-urban areas [39] respectively. All the included studies provided evidence on mHealth SRH information and services [29, 37–47]. The included studies were conducted in the following countries: two in Bangladesh [42, 43], three in Kenya [29, 38, 41], one in Ghana [46], one in Ghana and Malawi [39], one in Tanzania [37], one in Lesotho [40], one in Nigeria and Kenya [44], one in Uganda [47] and one in Cambodia [45]. Most studies (11) used qualitative method designs [29, 37–43, 45–47] with only one using mixed methods designs [44]. These studies involved male and female populations in community and health facility settings. All studies reported HCPs experiences on facilitators and barriers for delivering mobile phone based reproductive health services [29, 37–47].

### Synthesis of results

Data were analysed thematically. The synthesis included seven themes: Author and year, country and setting, study methods, study population, mHealth intervention platforms, barriers and facilitators detailed in Table 4.

**Table 4:** Summary of Studies included in the Systematic Review, *N* = 12

Author & year	Country& Setting	Study methods	Study population	mHealth intervention platforms	Barriers	Facilitators
Jahangir et al. [42]	Bangladesh Community-based Rural	Qualitative In-depth interviews	Health providers	Sexual health services SMS or text-messaging	- Low levels of technological and health literacy. -Not possible to provide diagnoses of STIs over the phone -Not possible to provide physical examination on phone. -Emotional burden for receiving too many calls and time.	-mHealth quite good for providing counselling. -Gets quick information to clients - Easy referral services to clinics - Time and cost management for traveling to health facilities. - Effective in time managemt for providing services. -Culturally appropriate in providing SRH information. -Effective in providing greater access to health information for women regarding STIs. -Provides an innovative platform to bridging the health communication gaps in sexual health
Peprah et al. [46]	Ghana Rural	Qualitative In-depth interviews	Healthcare providers	Sexual and reproductive services Phone call	-Language barrier. -Illiteracy or low educational level of recipients. - Lack of trust. -Mobile network connectivity challenges.	mHealth saves waiting hours’ time. -Delivering services via mHealth technology saves time. -mHealth reduces workload. -mHealth able to contact many clients at a time for healthcare.
Braun et al. [37]	Tanzania Rural	Qualitative IDIs	Community health workers	Family planning (FP) services text messaging	-Low technological skills. -Limited power for battery charging -Cost of mobile phone.	- Timelier - More convenient contacting clients from remote locations. -Ease of use of technology confidential information and care. -Increased method choice. -Improved privacy, confidentiality and trust with clients.
Dev et al. [38]	Kenya Rural	Qualitative In-depth interviews	Healthcare providers (nurses)	Contraception services Mobile phone call	- Limited technological literacy - Workload for receiving calls from clients. -Emotional stress.	-Helps deliver the appropriate and detailed SRH information. -Saves time for providing education or counselling. -Improves client provider interactions relationship. - Allows discuss confidential issues with women on contraceptives privately with women to make better decisions -Maximizes time or saves providers time in providing counselling process.
Logie et al. [44]	Nigeria Keyna Rural	Mixed method Qualitive In-depth interviews (IDIs)	Healthcare providers	Sexual and reproductive health services Mobile phone call	-Does not work if a client/ person doesn’t have a phone. -Lack of regular internet access. -Lack of technological literacy of using mobile apps. - Cost of mobile phones	-Able to target specific health information to clients in rural areas based on health demographics. -Able to provide access to SRH information for underserved group. - Easier to provide mHealth apps SRH confidentially for young people in remote areas. **-**Easy for healthcare providers to constantly remind or follow up on clients.
Ibembe [41]	Kenya Health facility Rural	Qualitative IDIs	Healthcare professionals	Reproductive health services Mobile phone call	-Lack of technological expertise. -Poor network connectivity. -Cost of phone credit or airtime. -Lack of motivation. -Lack of technological literacy and skills. - Not owning a cell phone.	-Easy consultation. - Addressing challenges distance between health providers and users. - Trust and confidentiality are built. Around health providers and users. -Quality and timely health decision making.
Ong et al. [45]	Cambodia Rural/urban	Qualitative Focus group discussions (FGDs) IDIs	Community health workers	Sexual and reproductive health/HIV Text messaging Voice messaging	-Lack of financial support for service provision. -Network connectivity interruptions.	- Able to provide information on HIV and STIs prevention issues. - Able to link up with many clients with SRH services. -Able to connect groups of clients. -Able to help clients in making SRH decisions -More efficient to deliver information directly and more frequently to a larger group via mobile phones.
Khatun et al. [43]	Bangladesh Rural	Qualitative IDIs		Health services Mobile phone call	-Technological human resource inadequacy. -Healthcare personnel readiness to use mobile technology for SRH services. -Lack of technological skills by some HCPs and young people. - illiteracy barriers.	-Decrease in patient loads in rural healthcare centers. -mHealth consultation saves time - Enables health providers to provide quality health services. -Culturally sensitive and technology friendly solution
Hirsch-Moverman et al. [40]	Lesotho Rural	Qualitative IDIs	Healthcare providers Health facility & community -based	Health services/HIV text messaging	-Lack of technology infrastructure. -Limited network connectivity -Limited electricity connectivity. -Community members influence of use of mobile phones for SRH services.	-Facilitates communication between patients’ providers. -Ability to be able to follow-up patients frequently. -mHealth communication messages strengthen the patient–provider bond. -Ability to monitor patients over phone. -Easily able to track and follow up patients.
Jennings et al. [29]	Kenya Rural	Qualitative FGDs/IDIs	Community health workers & nurses Health facility-based	HIV services Voice calls, Text messaging	- Cost for airtime for maintenance of phones. -Lack of technological by clients	- Protects the confidentiality of information. - Convenient for follow -Easy referral of clients to HCPs. -Timelier notification f information -Save time -Reduces unnecessary visits.to health facilities
Hampshire et al. [39]	Ghana & Malawi Peri-urban & rural	Qualitative IDIs	Health workers Community-based	Contraception/family planning/HIV prevention education. Text messaging phone calls Voice messaging	-Not having personal mobile phones. -Temporary mobile phone breakdown can be problematic. -Poor or unreliable network - Mobile phone credit or airtime. -Limited sources to buy credit Emotional burden for receiving calls at night.	--Mobile phones help in emergencies, staff making emergency calls. -Helps in communicating with patients’ colleagues, obtaining clinical advice.
Chang et al. [39]	Uganda Rural	Qualitative IDIs	Peer health workers	HIV services Text messaging Voice messaging	-Phone maintenance cost -Lack of power/electricity to charge phones. -Mobile phones theft .	-Facilitates task shifting. Time saving. -Facilitates exchange of information or communication between HCPs and patients and HCPs. -Improved peer health workers morale.

### mHealth SRH intervention services delivered by HCPs

In this review, all HCPs had experience in providing mobile phone based SRH information and services among populations across rural settings in LMICs. The participants used different mobile platforms for providing SRH and services including text messaging, voice messaging, interactive voice responses and phone calls. Most of the studies used text messaging for the delivery of SRH information and services on contraception, family planning and STIs and HIV prevention. Overall, mHealth SRH interventions provided for young people tended to be educational [29, 37–47]. All the studies reported on participants varied experiences and perceptions on providing mHealth SRH information and services for rural people across studies settings. All the studies reported HCPs varied experiences on barriers and facilitators for providing mobile phone based SRH information and services across study settings.

### Facilitators to mHealth SRH services provision

The review findings have provided insights into HCPs views and experiences on factors acting as facilitators for the provision of mHealth-based SRH services for people in rural areas of LMICs [29, 37–47]. Most HCPs were supportive of the mHealth application for helping to address some of the challenges of providing SRH information and services in rural areas. Participants reported that mobile phone technology helps make timelier communication of SRH information and services to clients in hard-to-reach rural areas [42], providing more convenient and better quality information with improved privacy, confidentiality and trust compared to face-to-face consultations [29, 37, 38, 41, 46].

Another advantage of mHealth was time efficiency, because multiple health information messages and services could be delivered to groups of people [29, 45, 47]. This was especially pertinent to text messaging platforms [37, 42]. There were also cost savings for both HCPs and clients because there was no need to travel to health facilities [39]. In addition, HCPs said using mobile phones made it possible to task shift some responsibilities to lower cadre of health workforce remotely [29, 37–39, 45]. HCPs also described that mHealth helped facilitate referrals and follow up on clients to HCPs in health facilities [40], and was user-friendly [43]. mHealth was reported as being effective in bridging SRH communication gaps [42] providing greater access to health information regarding STIs (especially for women) and facilitated culturally appropriate SRH information provision [42].

### Barriers to mHealth SRH services provision

Barriers to mHealth service provision mainly consisted of infrastructural challenges [40, 41, 43] including limited and unreliable network connectivity [39–41, 44, 45], limited power for charging mobile phones [37, 40, 47]. Additionally, personal factors such as the cost of mobile phones and mobile credit [37, 41, 44], limited vendors or outlets for purchasing mobile credit [39] technological and health literacy, and linguistics barriers [40, 41, 43, 46] were cited as a challenge to the delivery and uptake of SRH mHealth among young people in rural settings [41–43]. HCPs also noted the emotional burden and workload of making and receiving texts and calls to and from clients [38, 39, 42]. Also identified in this review was the influence of community members with ingrained in social norms, especially for women, hindering effective provision and uptake of services [40].

## Discussion

Mobile health interventions were found to have the potential to improve the provision and uptake of SRH services among populations in rural areas of LMICs [16, 17, 25, 27, 50]. mHealth interventions were found to connect rural people directly to HCPs of SRH services and information [16, 17, 25, 27, 50]. This review shed some light on the opportunities and challenges for providing mHealth SRH information and services to young rural people [51]. This review provides evidence on facilitators and barriers for delivering and improving rural access to mobile phone based SRH information and services in rural settings in LMICs [29, 37–47].

Overall, our findings showed that mHealth interventions can be useful to improve provision and uptake of SRH services across a broad range of services among rural people [39]. Study participants reported facilitators such as the convenience of using mobile phone to deliver a range of SRH information and services remotely and confidentially [29, 37–47] reducing fear and stigma associated with face-to-face SRH consultations aligned with quantitative findings [44]. Also, saving of travel time and costs for both HCPs and users were noted [37, 39, 47], in line with research [5, 52–54].

An important facilitator for providing mHealth was the ability to task shift by delegating duties or responsibilities to lower-level cadre health professionals [42, 47]. HCPs said task shifting helped improved time management and workload for them to perform critical and urgent duties [29, 37–39, 45]. Task shifting has been identified as a pragmatic response to health workforce shortages in rural settings in LMICs [55]. It is observed however that the burden of task shifting tends to fall disproportionally on HCPs with lower qualifications and volunteers, leading to work overload without corresponding remuneration [55, 56]. To maximize task shifting benefits without placing an undue burden on HCPs who are willing to undertake additional workload, appropriate compensation and training need to be considered, to ensure the sustainability of mHealth programs in rural settings in LMICs [39].

In this review, services were provided using voice messaging, phone calls, voice calls and SMS text-messaging [29, 37–47]. SMS texting was seen as the most preferred and efficient platform for delivering health information and services, due to the ability to transmit multiple health messages to groups of people at the same time remotely and confidentially [37, 40, 42, 46]. A preference for delivering health information via SMS text messages in rural populations in LMICs settings has been reported [57]. There is a growing interest for the preference of mHealth interventions platforms in LMICs for SRH information and services for rural population. There is the need for research to understand the benefits and preferences of mobile phone-based platforms for users with greater reach in rural areas especially among lower literate populations.

The review highlighted several challenges which hinder the effective delivery and uptake of mHealth SRH information and services among young people in rural contexts in LMICs.

These mainly included technological challenges which hindered the effective delivery of SRH mHealth services [29, 37–47]. The major barriers included a lack of technical skills [40, 41, 43, 46] and limited technological infrastructure [40, 41, 43]. These findings have been reported by studies in LMICs [16, 27, 58, 59]. The full realization of the full potential of mHealth SRH services will require investment in the development of technological infrastructure [46, 60] and building the capacity of HCPs and users to effectively use innovative mHealth for the delivery and uptake of SRH services for rural populations [18, 61, 62].

Personal barriers in terms of cost related to mobile phones and credit were cited by participants [37, 41, 44]. Several studies conducted in similar settings in LMICs have confirmed these findings [16, 17, 27, 63, 64]. In some instances, HCPs had to bear mobile phone expenses in order to be able to provide the services [39]. A qualitative study in rural South Africa has reported similar findings [19]. Personal cost of providing health delivery services in rural settings in LMICs constitutes a disproportionate share of cost for HCPs and poor young people with low incomes [39]. HCPs said that subsidizing the cost of mobile phones and call credit for rural health workers and the creation of a hotline dedicated to mHealth SRH services [65] in rural areas of LMICs is critical for delivery of SRH information and services among rural and remote populations [66–68].

Also reported as a personal barrier were technological and health literacy and linguistics barriers [40, 41, 43, 46]. Technological literacy is a skill needed to access digital technology, which is necessary for mHealth uptake. Studies have shown that low or limited literacy skills are more prevalent among rural populations and may disguise HCPs and clients ability to understand health information [69, 70]. This may make health education and communication with HCPs with clients not effective and could lead to poor health outcomes in rural settings [70]. In rural contexts, findings suggested that the involvement of linguistically diverse HCPs to work with clients may be needed in order to meet the diversity of clients that make up various populations [42].

Emotional burden and workload related to making and receiving too many calls for serving clients were also identified by HCPs as barriers to mHealth provision [38, 39, 42]. The training of more HCPs in Digital health technology to support the delivery of mHealth education could mitigate emotional burden and workload among HCPs. This could also help them to disseminate culturally appropriate and sensitive SRH information among populations in rural contexts in LMICs [42].

Participants identified infrastructural or contextual barriers to mHealth delivery [40, 41, 43] including lack or weak network connectivity [39–41, 44, 45], and lack of electricity to charge mobile phones [37, 40, 47]. To ensure strong internet connectivity, it is suggested that installation of fiber optic and free public Wi-Fi in central areas where rural people can access the internet can improve the speed and access to internet for services. Alternative power sources such as solar panels for charging phones would also help [66].

The influence of community members and ingrained in socio-cultural norms also impacted use of mHealth for SRH service delivery [40]. In rural settings in LMICs, the provision and uptake of SRH information and services among rural populations is ingrained in traditional social norms [42, 71]. Providing innovative mHealth based SRH information and services was identified as culturally sensitive and user-friendly but this was not always sufficient to overcome cultural barriers [43]. mHealth programs are becoming an integral part of reproductive programs in rural LMICs [25, 50], so investment in education of community members is needed to effectively address socio-cultural and sensitive barriers to service provision in rural contexts in LMICs.

Finally, despite the potential for mHealth interventions to be scalable and integrated in rural healthcare settings, programme managers, policy makers and implementers need to address individual and socio- cultural norms that act as barriers, as well as fill infrastructural gaps. This will require collaboration between governments, nongovernmental organizations and other stakeholders.

### Strengths and limitations

A strength of this study is that it gives a clear review of the practical experiences of HCPs on facilitators and challenges for providing mHealth SRH services in rural settings in LMICs. Another strength of this study is that it covered a period of two decades from the inception of mHealth to date. In addition, all primary studies included in this review underwent a rigorous methodological quality appraisal. A major limitation of this study is that only studies written and published in English were included.

## Conclusions

There have been few studies of mHealth on barriers and facilitators for improving population health in rural settings in LMICs. Our review found that implementing innovative mHealth based SRH services could bridge a service access gap of SRH information and services in rural areas of LMICs. Despite the advantages of this technology, several challenges associated with delivering mHealth need to be urgently addressed to enable scale-up and integration of sexual and reproductive mHealth into rural health systems. Our recommendations serve as references for improving on existing mHealth services and the implementation of future studies in rural LMICs. However, further research is needed to explore HCPs experiences on the effectiveness of using mobile phone communication platforms for delivering SRH information and services in rural settings in LMICs. Furthermore, it is likely that mHealth service barriers and facilitators vary by cultural and country setting, underscoring the need for more nuanced research in this area.

## Supplementary Information

**Additional file 1.**

## Data Availability

The datasets used and/or analysed during the current study are available from the corresponding author on reasonable request.
