# Supplementary material for: Perspectives of health care professionals’ on delivering mHealth sexual and reproductive health services in rural settings in low-and-middle-income countries: a qualitative systematic review
Source: BMC Health Serv Res. 2022 Sep 9;22:1141. doi: 10.1186/s12913-022-08512-2 (PMC9461099; doi:10.1186/s12913-022-08512-2)
Supplement: Supplementary file 1 — Additional file 1. [file 12913_2022_8512_MOESM1_ESM.docx]

**Online Resource: Search strategies**

| **Cochrane Library** | |
| --- | --- |
| **1** | Trials matching young adult* OR youth* OR adolescent* OR young people* OR youth population* OR young wom?n* OR young girl* OR young boy* OR young m?n* OR young women* emerging adult* OR adolescent girl* OR adolescent boy* OR adolescen |
| **2** | in Title Abstract Keyword AND reproductive health* OR sexual health* OR HIV* OR contraception* OR contraceptives* OR modern contraception* OR contracept service* OR contracept educat* OR contracept counsell |
| **3** | in Title Abstract Keyword OR Healthcare providers* OR Healthcare professionals* OR health provider* OR health counsellor* OR health educator |
| **4** | in Title Abstract Keyword AND mobile health* OR mHealth* OR mobile phone health technology* OR mobile phone health* OR digital mobile health* OR digital mobile phone health |
| **5** | in Title Abstract Keyword AND low-income countries* OR low-and-middle-income nation* OR low to middle income countries* OR middle-income countr* OR low resource countries |
| **6** | in Title Abstract Keyword - with Cochrane Library publication date Between Jan 2000 and Dec 2020, in Trials with 'Public Health' in Cochrane Groups (Word variations have been searched) |
| **Scopus** | |
| **1** | (TITLE-ABS-KEY (low-income AND countries* OR low-and-middle income AND nation*  OR  low  AND to  AND middle  AND income  AND countries*  OR  middle-income  AND countr*  OR  low  AND resource  AND countries )  AND |
| **2** | TITLE-ABS KEY (mobile AND health* OR mhealth* OR mobile AND phone AND health AND technology* OR mobile AND phone AND health* OR digital AND mobile AND health* OR digital AND mobile AND phone AND health) OR |
| **3** | TITLE-ABS-KEY (healthcare AND providers* OR healthcare AND professionals* OR health AND provider* OR health AND counsellor* OR health AND educator ) OR |
| **4** | TITLE-ABS-KEY (young AND adult* OR youth* OR adolescent* OR young AND people* OR youth AND population* OR young AND wom?n* OR young AND girl* OR young AND boy* OR young AND m?n* OR young AND women* AND emerging AND adult* OR adolescent AND girl* OR adolescent AND boy* OR adolescen ) OR |
| **5** | TITLE-ABS-KEY (reproductive AND health* OR sexual AND health* OR HIV* OR contraception* OR contraceptives* OR modern AND contraception* OR contracept AND service* OR contracept AND educat* OR contracept AND counsell ) ) |
| **6** | AND PUBYEAR > 1999 AND PUBYEAR < 2021. |
| **CINAHL** | |
| S4 | ( reproductive health* AND sexual health* AND HIV* AND contraception* AND contraceptives* AND modern contraception* AND contracept service* AND contracept educat* AND contracept counsell* AND young adult* AND youth* AND adolescent* AND young people* AND youth population* AND young wom?n* AND young girl* AND young boy* AND young m?n* AND young women* AND adolescent girl* AND adolescent boy* AND adolescen ) OR ( Healthcare providers* AND Healthcare professionals* AND health provider* AND heal ... |
| S3 | (reproductive health* OR sexual health* OR HIV* OR contraception* OR contraceptives* OR modern contraception* OR contracept service* OR contracept educat* OR contracept counsel* OR young adult* OR youth* OR adolescent* OR young people* OR youth population* OR young wom?n* OR young girl* OR young boy* OR young m?n* OR young women* emerging adult* OR adolescent girl* OR adolescent boy* OR adolescen Limiters - |
| S2 | ( reproductive health* OR sexual health* OR HIV* OR contraception* OR contraceptives* OR modern contraception* OR contracept service* OR contracept educat* OR contracept counsel* OR young adult* OR youth* OR adolescent* OR young people* OR youth population* OR young wom?n* OR young girl* OR young boy* OR young m?n* OR young women* emerging adult* OR adolescent girl* OR adolescent boy* OR adolescen ) AND ( mobile health* OR mHealth* OR mobile phone health technology* OR mobile phone health* OR ... |
| S1 | ( reproductive health* OR sexual health* OR HIV* OR contraception* OR contraceptives* OR modern contraception* OR contracept service* OR contracept educat* OR contracept counsel* OR young adult* OR youth* OR adolescent* OR young people* OR youth population* OR young wom?n* OR young girl* OR young boy* OR young m?n* OR young women* emerging adult* OR adolescent girl* OR adolescent boy* OR adolescen ) AND ( mobile health* OR mHealth* OR mobile phone health technology* OR mobile phone health* OR ... |
|  | Limiters - Full Text; References Available; Published Date: 20000101-20201231; Peer Reviewed; Clinical Queries: Qualitative - Best Balance; Human; Journal Subset: Public Health; Geographic Subset: Africa; Language: English; Special Interest: Men's Health, Public Health, Women's Health |
| **PsychoINFO** | |
| 1 | (young adult* or youth* or adolescent* or young people* or youth population* or young wom?n* or young girl* or young boy* or young m?n* or young women* emerging adult* or adolescent girl* or adolescent boy* or adolescen).mp. [mp=title, abstract, heading word, table of contents, key concepts, original title, tests & measures, mesh] |
| 2 | (reproductive health* or sexual health* or HIV* or contraception* or contraceptives* or modern contraception* or contracept service* or contracept educat* or contracept counsell).mp. [mp=title, abstract, heading word, table o’f contents, key concepts, original title, tests & measures, mesh] |
| 3 | (Healthcare providers* or Healthcare professionals* or health provider* or health counsellor* or health educator).mp. [mp=title, abstract, heading word, table of contents, key concepts, original title, tests & measures, mesh] |
| 4 | (mobile health* or mHealth* or mobile phone health technology* or mobile phone health* or digital mobile health* or digital mobile phone health).mp. [mp=title, abstract, heading word, table of contents, key concepts, original title, tests & measures, mesh] |
| 5 | (low-income countries* or low-and-middle-income nation* or low to middle income countries* or middle-income countr* or low resource countries).mp. [mp=title, abstract, heading word, table of contents, key concepts, original title, tests & measures, mesh] |
| 6 | limit to (full text and human and english language and yr="2000 - 2020") |
